# Mechanisms of ulnar collateral ligament injury in baseball: criteria and rationale for return to play — a systematic review

**DOI:** 10.1186/s13102-025-01499-3

**Published:** 2025-12-27

**Authors:** Di Huang, William J. Lew, Brent Shimoda, Eli M. Snyder, Kai Sheng Khor, David X. Cifu, Henry L. Lew

**Affiliations:** 1 Johns Hopkins Bloomberg School of Public Health, Baltimore, MD USA; 2 Hualien Tzu Chi Hospital, Buddhist Tzu Chi Medical Foundation, Hualien, Taiwan; 3 John A. Burns School of Medicine, University of Hawaii, Honolulu, HI USA; 4 Department of Physical Medicine and Rehabilitation, Johns Hopkins University School of Medicine, Baltimore, MD USA; 5 Department of Physical Medicine and Rehabilitation, Virginia Commonwealth University, Richmond, VA USA; 6 Office of Medical Education, John A. Burns School of Medicine, University of Hawaii, Honolulu, HI USA

**Keywords:** Ulnar collateral ligament, Return to play, Baseball injuries, Throwing athletes, Rehabilitation criteria

## Abstract

**Background and aims:**

Return-to-play (RTP) readiness is a critical consideration for common baseball injuries, including after ulnar collateral ligament (UCL) injuries. UCL injuries, often resulting from repetitive throwing stress, can significantly impact an athlete’s career, requiring interventions ranging from nonoperative rehabilitation to surgical reconstruction. While RTP criteria for these injuries exist, they remain inconsistent across studies and clinical practice. Commonly reported RTP criteria include achieving pain-free range of motion and completion of progressive throwing distances. This systematic review aims to analyze and compare different RTP criteria for UCL injuries in baseball players.

**Methods:**

This systematic review follows the PROSPERO protocol (CRD42024607322) and adheres to the PRISMA guidelines. Three independent reviewers conducted a comprehensive search across PubMed, Scopus, and Embase to identify relevant studies. Search results were imported into Covidence for systematic screening and data management. Any disagreements or uncertainties in study selection were resolved by a fourth reviewer to ensure consistency and accuracy. After full-text screening and eligibility assessment, 18 articles were included for data extraction.

**Results:**

Eighteen studies on UCL injuries (*n* = 2887) were included. UCL injuries predominantly affected professional or collegiate pitchers in baseball and were linked to valgus stress during high-velocity throwing. Reported rehabilitation protocols varied, ranging from brief immobilization followed by progressive range-of-motion and throwing programs to structured three-month conservative interventions before considering surgery. RTP was assessed using objective performance metrics such as earned run average (ERA), walks and hits per inning pitched (WHIP), and advanced metrics like Wins Above Replacement (WAR). RTP rates ranged from 80% to 91%, with most players returning to their pre-injury level of competition. Some studies reported incomplete protocol adherence due to pain, lack of motivation, or pursuit of alternative treatments.

**Conclusion:**

This review highlights inconsistencies and differences in RTP criteria for UCL injuries. Key influencing factors include treatment type, competition level, and psychological readiness. Although metrics such as ERA, WHIP, and imaging findings are frequently used, their application lacks uniformity. Standardized, multidisciplinary RTP protocols integrating physical, imaging, and psychological components are essential to guide safe and objective return-to-sport decisions.

**Supplementary Information:**

The online version contains supplementary material available at 10.1186/s13102-025-01499-3.

## Introduction

Ulnar collateral ligament (UCL) injuries are not uncommon in baseball and can have lasting impacts on a player’s career. Often caused by repetitive throwing stress from pitching, UCL stress can result in ligamentous damage that compromises throwing ability [1]. This valgus stress on the elbow during the acceleration phase of pitching can reach up to 64 N-m, surpassing the UCL’s threshold of tensile strength [2]. As a result, nearly 15% of minor league and 25% of Major League Baseball (MLB) pitchers have undergone ulnar collateral ligament reconstruction (UCL-R) by the time they reach the professional level [3]. Depending on the injury severity, treatment options of UCL injuries range from nonsurgical management to surgical intervention [4]. Determining return-to-play (RTP) readiness is an integral component of sports medicine care and the use of standard RTP guidelines for common injuries are important to efficiently reintegrate athletes back to play as RTP decisions play a key role in not only performance but long-term health outcomes. Thus, an evidence-based RTP protocol for UCL injuries would ensure that players recover fully before resuming play to avoid premature return and increased risk of recurring injury. The goal of this systematic review is to summarize existing RTP criteria for UCL injuries in baseball, emphasizing the distinct recovery needs and differences. By exploring the injuries’ characteristics and rehabilitation processes and challenges, we seek to support the development of consistent, tailored RTP practices to promote safer and more effective decision-making for UCL injuries.

## Method

### Search strategy and study eligibility

This systematic review was registered on PROSPERO under the registration number CRD42024607322. Three independent reviewers (D.H., K.S.K., W.L.) conducted a comprehensive search across Medline (PubMed), Scopus, and Embase following the PRISMA statement to identify relevant studies. All databases were last searched on October 29, 2024. A combination of MeSH terms: *Ulnar collateral ligament injury*,* return to play*,* and baseball*, was set to maximize sensitivity. We also conducted a manual search of the reference lists from all included articles to identify any additional eligible studies. Three independent reviewers (D.H., K.S.K., W.L.) selected articles through abstract, title and full-text review. Any disagreements between the reviewers were resolved through consultation with a fourth reviewer (B.S.). The initial search strategy was developed by the first author (D.H.) and subsequently refined through critical input and revisions from the multidisciplinary team of researchers with expertise in systematic review methodology. Studies were included if they (1) involved baseball players of any competitive level diagnosed with UCL injury; (2) reported RTP criteria, protocols, or outcomes following UCL injury; and (3) were original human research studies, published in peer-reviewed journals. Eligible study designs included randomized controlled trials, cohort studies (prospective or retrospective), case–control studies, and case series. Studies were excluded if they (1) were reviews, meta-analyses, editorials, letters, single case reports, or conference abstracts; (2) did not report RTP outcomes or criteria; or (3) were non-baseball populations or non-English publications where translation was not possible. No restrictions were placed on publication date or language. Search results were imported into Covidence for systematic screening [5]. The review protocol was conducted between October and November 2024. The complete search strategy, including all keywords and search terms, is provided in Supplementary Appendix 1.

The research question was developed according to the Population, Intervention, Comparator, and Outcomes (PICO) framework. The population consisted of baseball players at any level who sustained UCL injuries. The intervention included RTP criteria and protocols (throwing-program milestones, time-based gates; operative or non-operative pathways). The comparator involved alternative RTP criteria or different management approaches (surgery type, rehabilitation models). The outcomes evaluated included RTP rate, time to RTP, return to previous level of play, post-RTP performance indicators.

## Data extraction

Two reviewers (D.H., K.S.K.) independently extracted data from each study using a standardized data extraction, developed and approved by the research team. Any disagreements between the reviewers were resolved through consultation with a third reviewer (B.S.). The extracted information included: (1) study characteristics such as geographic location, published years, sample size; (2) participant demographics, including mean age; (3) outcomes, categorized into primary outcomes observable on the baseball field such as pitching velocity, earned run average (ERA), innings pitched, and batting performance statistics (e.g., on-base percentage, slugging percentage). These measures reflected the athlete’s ability to resume prior performance levels following UCL injury or reconstruction. Secondary outcomes typically assessed by physicians, such as ultrasound findings, MRI, and subjective pain or functional scores (e.g., KJOC) These outcomes provided complementary information about physiological recovery and structural healing; and (4) details and challenges of the return-to-play (RTP) protocols described in each study.

## Quality assessment

Risk of bias for all included studies was assessed using the Risk of Bias in Non-Randomized Studies of Interventions (ROBINS-I Version 2.0) tool [6]. This instrument was selected to ensure a consistent and comprehensive assessment framework across diverse study designs. Each domain was assessed independently by two reviewers (D.H., W.L.) and categorized as low, moderate, serious, or critical risk of bias. Per ROBINS-I guidance, a study with one or more domains rated at “critical” risk was considered to have an overall critical risk of bias. Any discrepancies between the reviewers were resolved through consultation with a third reviewer (B.S.).

## Results

### Study selection

**Fig. 1:**
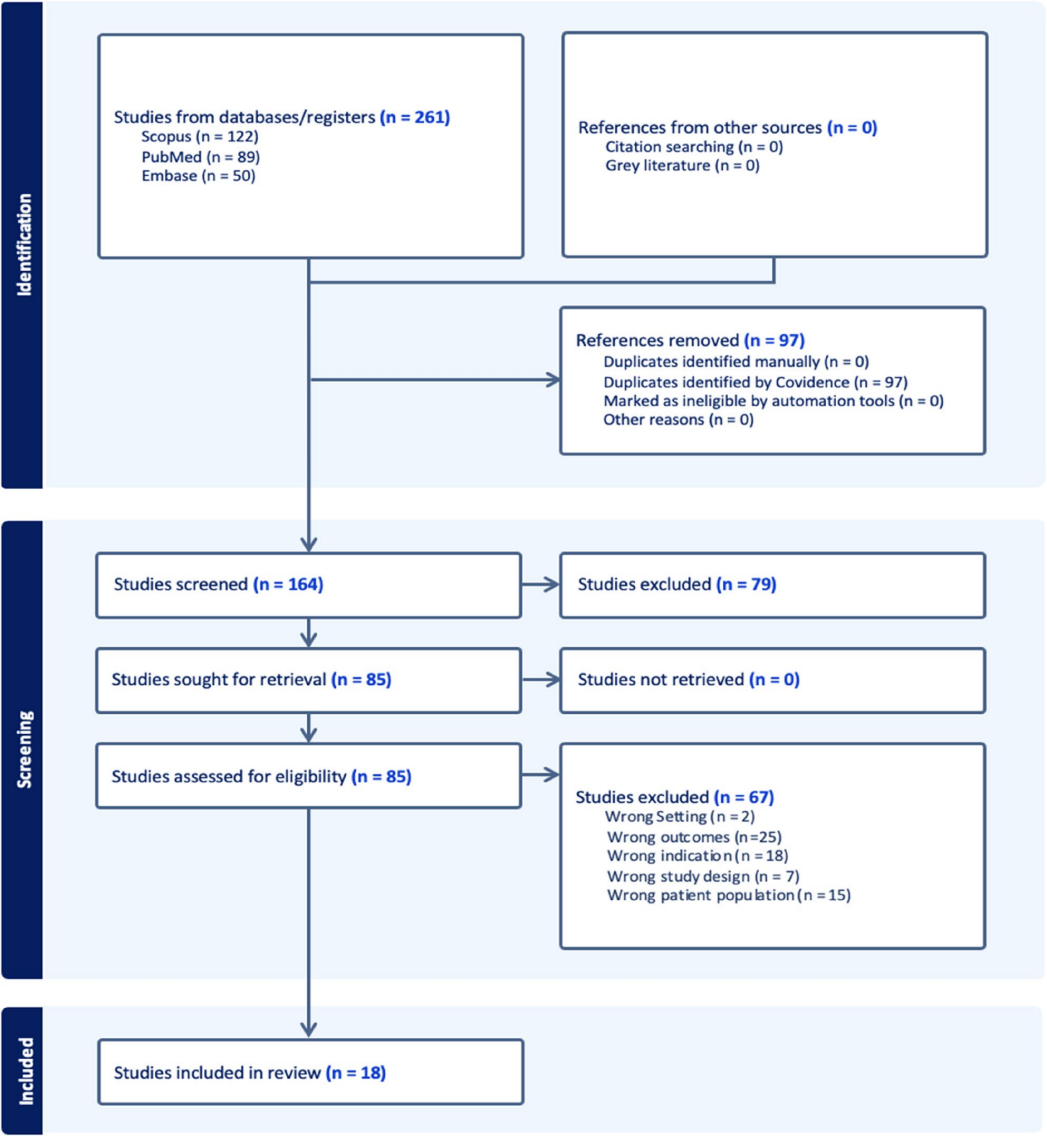
PRISMA flowchart

A total of 261 studies were initially identified through searches of PubMed, Scopus, and Embase. After removing duplicates, 164 articles remained for title and abstract screening. Based on predefined inclusion criteria and through full text review, 18 studies were ultimately included for data extraction [7–24]. The study selection process is summarized in Fig. 1.

### Quality assessment

Of the 18 included studies, 4 studies were graded as having an overall moderate risk of bias [19, 21–23], 6 studies were rated as having a serious risk of bias [7, 10, 13–15, 24], and 8 studies were rated at critical risk of bias [8, 9, 11, 12, 16–18, 20]. 

**Fig. 2:**
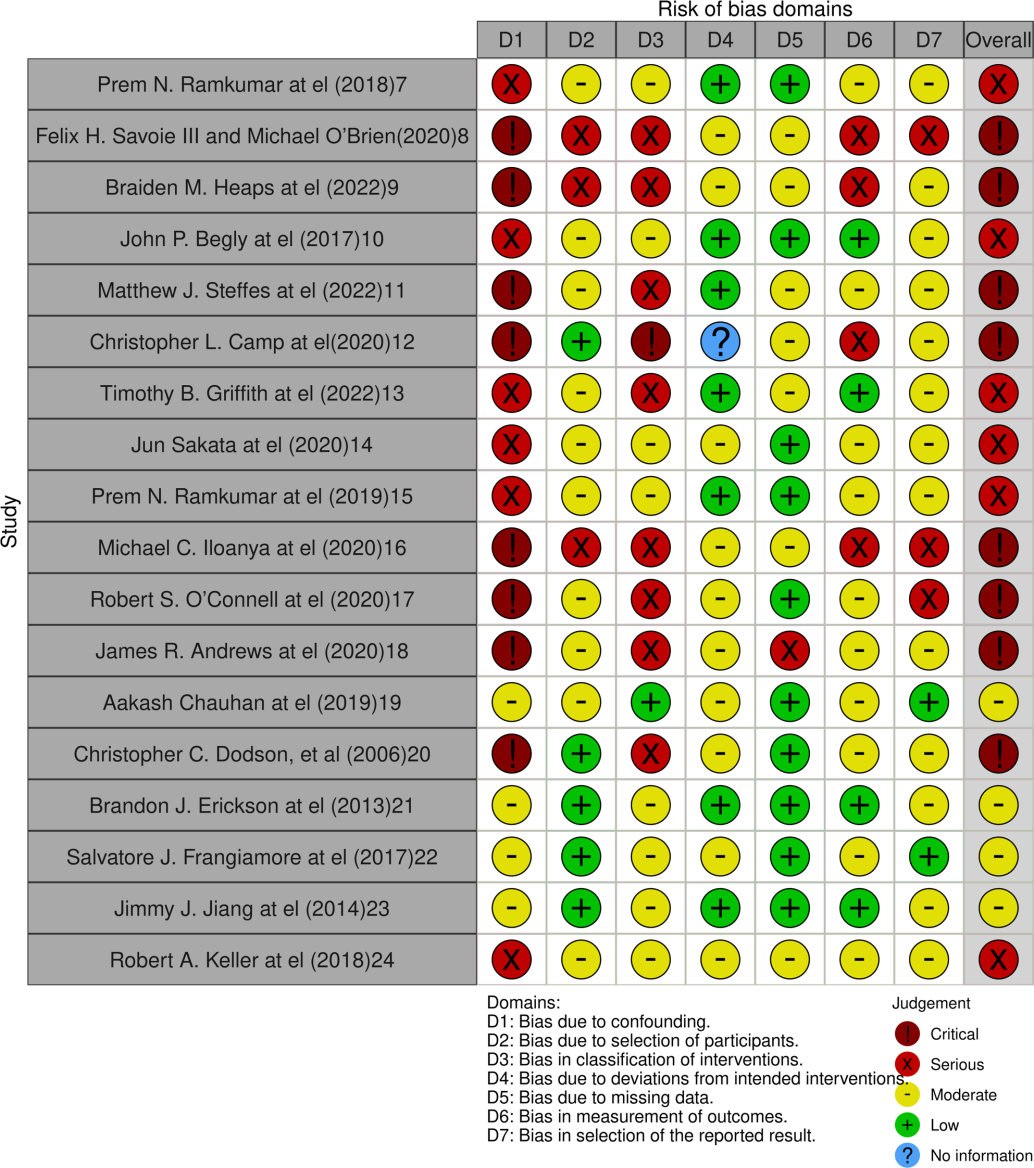
Summary of Risk of Bias Assessment

The most common sources of bias were confounding by indication, lack of a comparator group, selective inclusion or follow-up, and non-blinded outcome measurement. While several studies used matched control groups or multivariable analysis to address confounding, most remained observational and retrospective in nature, limiting causal inference. Many single-arm case series lacked adjustment, objective outcome verification, or protocol pre-registration. A detailed summary of ROBINS-I assessments by domain for each study is visualized in Fig. 2, generated using the *robvis* visualization tool [25].

### Findings

Data from eighteen eligible studies with a total of 2887 baseball players were included for UCL injuries. All selected studies focused on professional or collegiate-level baseball players, primarily pitchers, reflecting a high-performance athletic group particularly vulnerable to valgus overload and elbow instability. Mechanism of the injury was reported in eight papers [9, 10, 13, 16, 19–21, 23]: The UCL, especially its anterior bundle, is the primary stabilizer of the elbow against valgus stress, bearing up to 55% of the load during throwing [23]. Repetitive high-velocity motions, such as pitching, generate forces up to 290 N and torques of 64 Nm, leading to microtrauma and potential UCL insufficiency [13]. Rehabilitation processes varied across studies. Some outlined detailed protocols: for example, one study described a structured 3-month rehabilitation period prior to considering surgical intervention [7], while another emphasized immobilization in a posterior slab splint for up to 10 days followed by progressive range-of-motion and throwing programs [8]. Among included studies, several did not describe detailed rehabilitation or RTP protocols but provided data on RTP timing or outcomes [9, 10, 16, 19, 21–23]. 

We categorized the RTP of the UCL studies by objective, performance-based primary outcome measures, such as Earned Run Average (ERA), walks and hits per inning pitched (WHIP), innings pitched, win-loss records, and advanced sabermetrics like Wins Above Replacement (WAR) and Isolated Power (ISO). These primary outcome measurements serve as quantifiable indicators of performance recovery. One research used a range of pitching metrics, including ERA and win percentage, to assess post-treatment performance [11], whereas others reported ERA and strikeout counts [9, 21]. Secondary outcome measures, including MRI-based ligament grading systems and dynamic ultrasonography, offered structural confirmation; however, these were reported less consistently in the RTP process and were often subject to individual interpretation. The summarized results are presented in Tables 1 and 2, while the detailed data are provided in Supplementary Appendix 2.

**Table 1:**
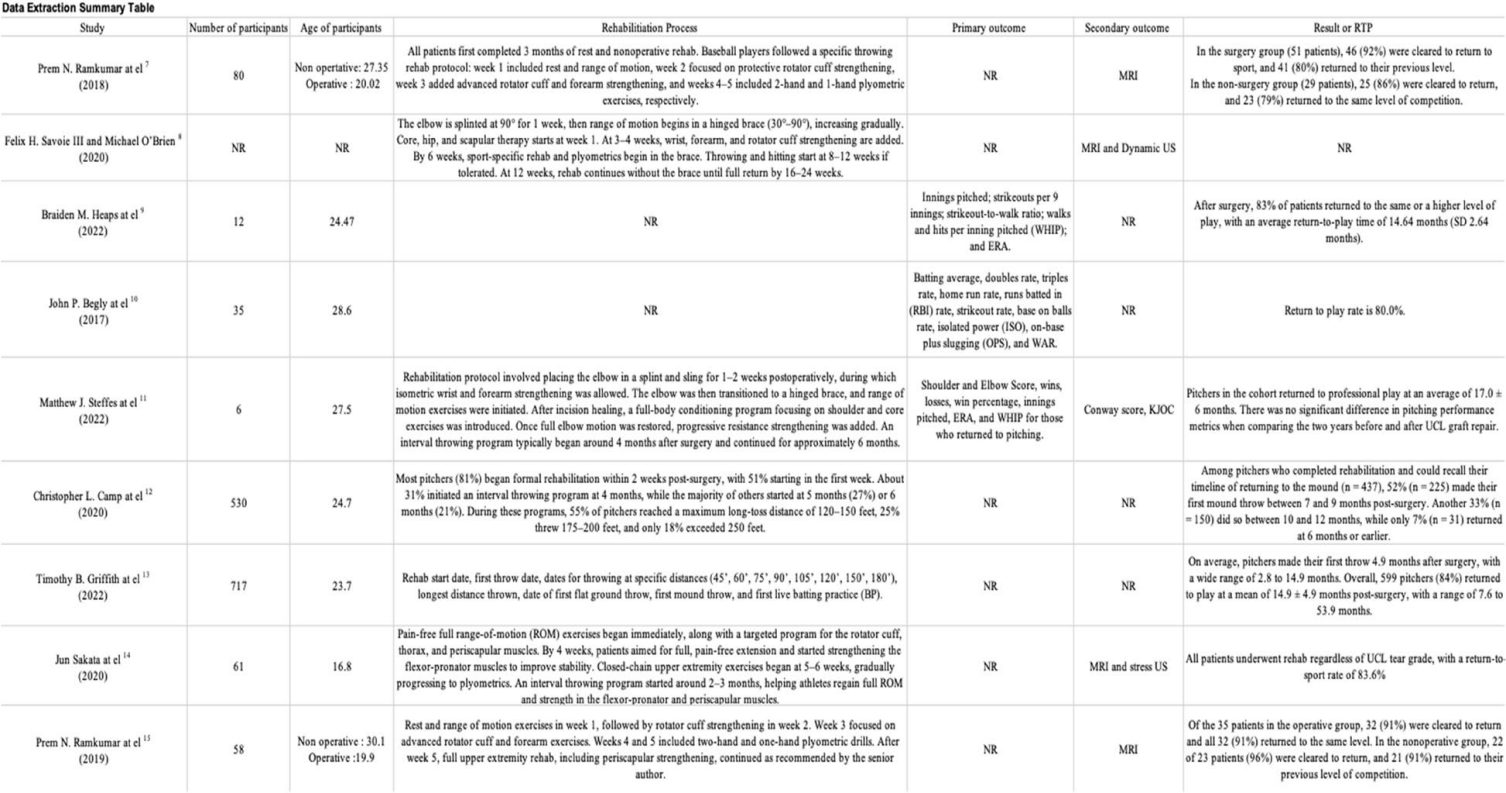
Data extraction summary [7–15]

**Table 2:**
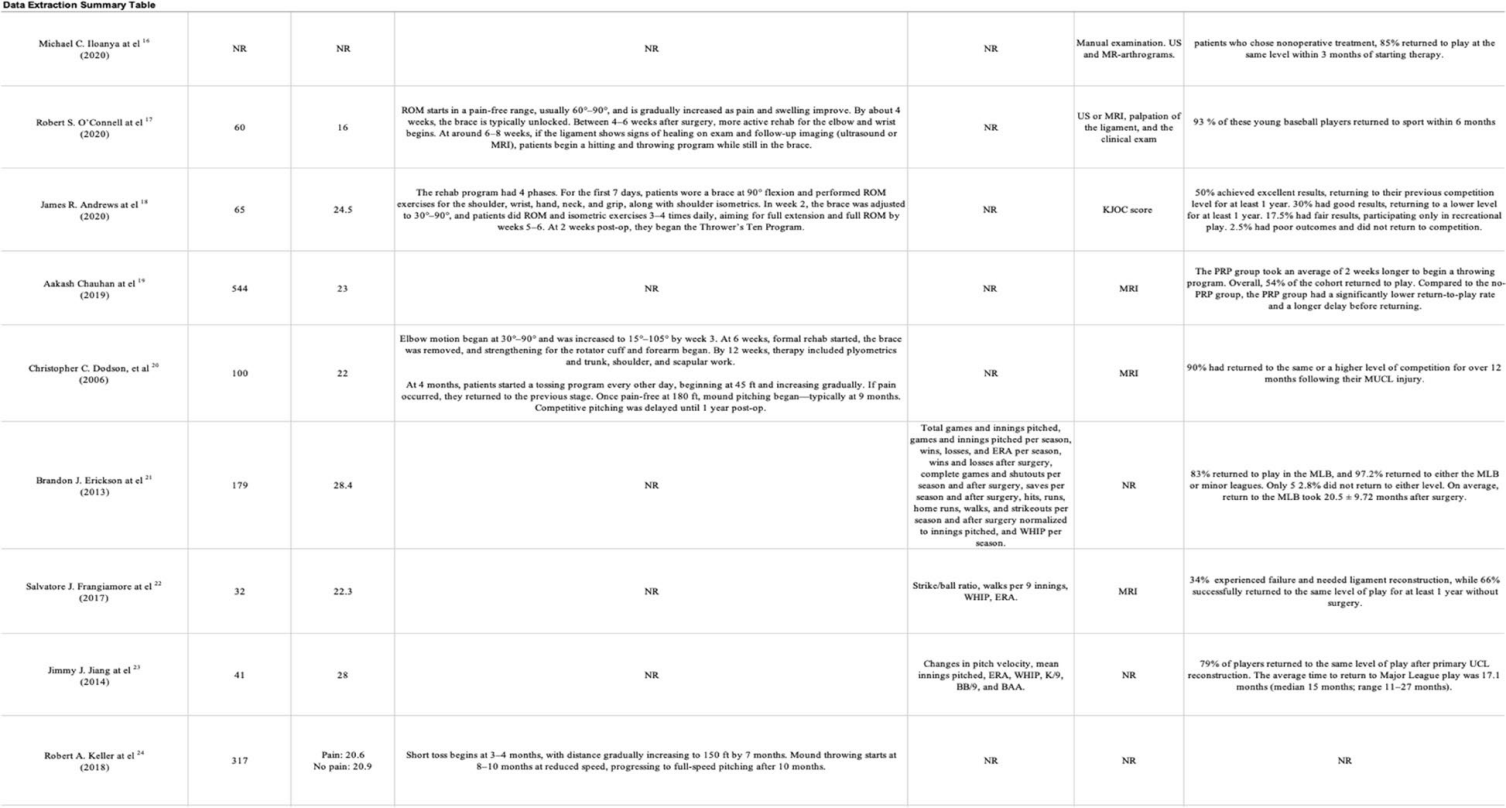
Data extraction summary [16–24]

Return-to-play outcomes were generally favorable for UCL injuries. Studies reported RTP rates between 80% and 91% for UCL injury with most athletes returning to their previous level of competition [10, 15]. Some studies highlighted the challenges of adherence to RTP protocols, as players withdrew due to pain, loss of interests, and seeking alternative treatments [7, 15, 22].


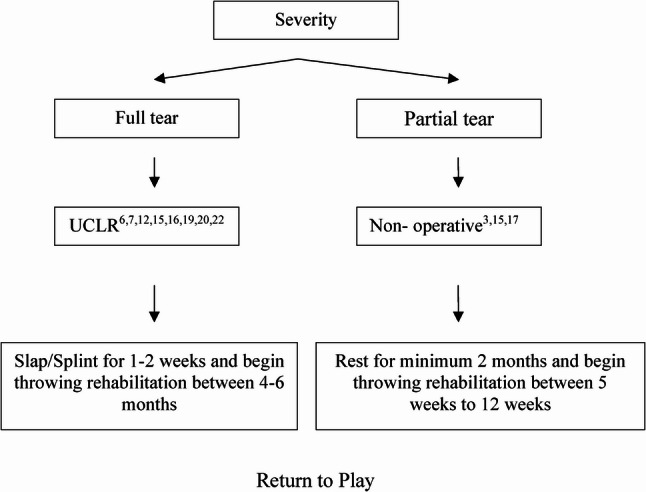


## Discussion

Our systematic review highlights the complex and variable RTP processes associated with UCL injuries. UCL injuries are one of the most common injuries in baseball [26]. The condition can significantly impact players’ availability and performance, and our review focuses on their injury mechanisms, differences in evaluation criteria, and rehabilitation approaches. UCL injuries, caused by biomechanical overload in the throwing arm, could be relatively measured by structured and performance-oriented assessment tools. Metrics such as ERA, WHIP, and WAR are commonly utilized as a relatively objective assessment for post-injury performance and were consistently used in the RTP protocols [27]. However, despite these standardized outcome measures, there were considerable differences in rehabilitation strategies, suggesting that these metrics alone may not adequately capture the complexity of recovery outcome. UCL RTP protocols ranged from short-term immobilization to phased return-to-throwing programs, with some studies providing detailed guidance whereas others lacked clarity. Furthermore, inconsistent use of imaging as a secondary outcome, no consensus could be made to link structural recovery with the timing for players to return to play. This variability suggests that while RTP after UCL injury is often guided by measurable performance indicators, the lack of consensus regarding optimal rehabilitation timelines and standardized criteria makes it challenging to provide precise recommendations or establish universally applicable guidelines.

The management of UCL injuries typically classified into non-surgical and surgical treatments. Non-surgical treatments, which include rest, physical therapy, and, in some cases, biologic injections such as platelet-rich plasma (PRP) were generally recommended for partial UCL tears or athletes with lower performance demands [28, 29]. These approaches aimed to restore ligament stability and function without surgery. The reported success rate for non-surgical treatments ranges from 87% to 100%, particularly when appropriate rehabilitation and return-to-play protocols were followed [28]. 

The “Tommy John procedure” has remained the standard surgical reconstruction technique for complete UCL tears [30]. This procedure consistently demonstrated high RTP rates ranging from 77% to 85%, for MLB pitchers, although definitions and metrics for return to play varied across studies [31]. In recent years, UCL repair with internal bracing emerged as a viable alternative to full ligament reconstruction [32]. This alternative surgical treatment was particularly suited to proximal or distal avulsion-type UCL injuries with good ligament quality. This technique preserves the native ligament and reinforces it using an internal synthetic brace, allowing for faster rehabilitation and earlier RTP timelines. Early studies reported higher satisfaction rate with reduced recovery periods [33]. However, comprehensive long-term outcomes and direct comparisons with traditional reconstruction techniques had not yet been fully studied.

The athlete’s level of competition was another factor identified as contributing to the variability in RTP data. Professional athletes often have access to superior medical care, individualized rehabilitation, and additional external motivators, such as future contracts, sponsorships, and increased visibility, all of which could influence both the decision to undergo surgery and the speed of returning to play [34]. In contrast, collegiate or youth athletes often followed more conservative timelines or chose to forgo surgery altogether. Furthermore, the younger athletes frequently depended on family support to bear the financial burden of surgical procedures and postoperative rehabilitation. These economic factors likely influenced the decisions regarding RTP implementation as well. A systematic review indicated that younger athletes who undergo UCL reconstruction returned to play at a high rate [35]; however, concerns persisted regarding the potential for recurrent injury or ulnar nerve complications in this population. Of note, psychological factors such as fear of reinjury, loss of interest, or lack of confidence in the repaired ligament could delay or even prevent a full return to play, despite the absence of physical limitations [36]. Therefore, incorporating psychological assessments into rehabilitation protocols may enhance overall recovery outcomes.

### Limitations

Many studies failed to report the use of validated or standardized frameworks, and only a few incorporated comprehensive multidisciplinary assessments involving both physical evaluations and clinical expertise in the RTP protocol for UCL injuries. These limitations made a comprehensive analysis of these studies impossible. Sport medicine physicians should recognize that effective RTP protocol for UCL injuries extends beyond addressing visible structural damage alone, and should encompassed psychological, financial, and socio-environmental dimensions as well. Another limitation is the use of a single risk-of-bias tool (ROBINS-I) for all study designs, including single-arm studies. Although this approach ensured consistency, certain study types might have been more appropriately appraised using JBI or NIH instruments. Another limitation is the heterogeneity and incomplete reporting of rehabilitation protocols across studies. Many studies reported RTP outcomes without specifying the underlying criteria or progression milestones, limiting direct comparisons of protocol efficacy.

## Conclusions

RTP following UCL injuries remains a critical issue to address as it directly impacts player safety, performance, and career longevity. Despite increased standardization of RTP criteria, significant variability persists in evaluation methods and rehabilitation management strategies. The absence of standardized rehabilitation protocols further complicates clinical guidance, potentially compromising the consistency and adequacy of patient care. Therefore, developing validated and structured rehabilitation management protocols, along with standardized outcome criteria, is essential for optimizing recovery outcomes and successfully returning baseball players to competitive play following UCL injuries.

## Supplementary Information

Supplementary Material 1.

Supplementary Material 2.

Supplementary Material 3.

## Data Availability

All data generated or analyzed in this review are included in this published article and its supplementary information files. The detailed search strategy and extracted data tables are provided in the Supplementary Appendices.

## Abbreviations

RTP: Return to play
UCL: Ulnar collateral ligament
UCL-R: Ulnar collateral ligament reconstruction
ERA: Earned run average
WHIP: Walks and hits per inning pitched
WAR: Wins above replacement
MRI: Magnetic resonance imaging
