# Supplementary material for: Mechanisms of ulnar collateral ligament injury in baseball: criteria and rationale for return to play — a systematic review
Source: BMC Sports Sci Med Rehabil. 2025 Dec 27;18:38. doi: 10.1186/s13102-025-01499-3 (PMC12853774; doi:10.1186/s13102-025-01499-3)
Supplement: Supplementary file 1 — Supplementary Material 1. [file 13102_2025_1499_MOESM1_ESM.docx]

Medline (Pubmed):

("Ulnar collateral ligament" OR "UCL injury" OR "Elbow ligament injury" OR "Elbow injury")) AND (("Return to Sport"[Mesh] OR "Recovery of Function"[Mesh] OR "Rehabilitation"[Mesh] OR "Physical Therapy Modalities"[Mesh] OR "Treatment Outcome"[Mesh] OR "Decision Making"[Mesh] OR "Guideline Adherence"[Mesh] OR "return to play" OR "return to sport" OR RTP OR rehabilitation OR management OR recovery OR treatment OR "return to competition") AND (criteria OR guidelines OR protocols OR "decision making" OR rationale))) AND ("Throwing" OR "Baseball" OR "Overhead athlete")

Embase:

('Ulnar collateral ligament injury'/exp OR 'ulnar collateral ligament':ti,ab OR 'ucl injury':ti,ab OR 'elbow injury'/exp OR 'elbow ligament injury':ti,ab OR 'elbow injury':ti,ab) AND ('return to sport'/exp OR 'return to play':ti,ab OR 'functional recovery'/exp OR 'rehabilitation'/exp OR rehabilitation:ti,ab OR 'physical therapy'/exp OR 'treatment outcome'/exp OR 'decision making'/exp OR 'guideline adherence'/exp OR management:ti,ab OR recovery:ti,ab OR treatment:ti,ab OR 'return to competition':ti,ab) AND (criteria:ti,ab OR guidelines:ti,ab OR protocols:ti,ab OR 'decision making'/exp OR rationale:ti,ab) AND ('throwing'/exp OR throwing:ti,ab OR 'baseball'/exp OR baseball:ti,ab OR 'overhead athlete':ti,ab)

Scopus:

('ulnar collateral ligament injury'/exp OR 'ulnar collateral ligament':ti,ab OR 'ucl injury':ti,ab OR 'elbow injury'/exp OR 'elbow ligament injury':ti,ab OR 'elbow injury':ti,ab) AND ('return to sport'/exp OR 'return to play':ti,ab OR 'functional recovery'/exp OR 'rehabilitation'/exp OR rehabilitation:ti,ab OR 'physical therapy'/exp OR 'treatment outcome'/exp OR 'decision making'/exp OR 'guideline adherence'/exp OR management:ti,ab OR recovery:ti,ab OR treatment:ti,ab OR 'return to competition':ti,ab) AND ('throwing'/exp OR throwing:ti,ab OR 'baseball'/exp OR baseball:ti,ab OR 'overhead athlete':ti,ab)
